# Increased adipose tissue expression of TLR8 in obese individuals with or without type-2 diabetes: significance in metabolic inflammation

**DOI:** 10.1186/s12950-016-0147-y

**Published:** 2016-12-08

**Authors:** Rasheed Ahmad, Shihab Kochumon, Reeby Thomas, Valerie Atizado, Sardar Sindhu

**Affiliations:** 1 Immunology & Innovative Cell Therapy Unit, Dasman Diabetes Institute (DDI), Al-Soor Street, P.O. Box 1180, Dasman, 15462 Kuwait; 2 Tissue Bank Core Facility, Dasman Diabetes Institute (DDI), Al-Soor Street, P.O. Box 1180, Dasman, 15462 Kuwait

**Keywords:** Adipose tissue, Metabolic inflammation, Obesity, TLR8, Type-2 diabetes

## Abstract

**Background:**

The innate immune Toll-like receptors (TLRs) 2/4 are important players in chronic low-grade inflammation called metabolic inflammation in obesity and type-2 diabetes (T2D). While TLR2/4 expression changes associated with metabolic inflammation are known, the adipose tissue expression of endocytic TLR8, which is expressed by all major macrophage subsets, remain unclear. We, therefore, determined the TLR8 mRNA/protein expression in the adipose tissue samples from lean, overweight, and obese individuals with or without T2D.

**Methods:**

Subcutaneous fat biopsy samples were collected from 49 non-diabetic (23 obese, 17 overweight, and nine lean) and 45 T2D (32 obese, ten overweight, and three lean) individuals. TLR8 gene expression was determined using real-time RT-PCR and TLR8 protein expression was assessed by both immunohistochemistry and confocal microscopy. The changes in TLR8 expression were compared with those of macrophage markers, proinflammatory cytokines/chemokines, and surface TLRs/adapter proteins. The data were analyzed using *t*-test/Mann-Whitney *U*-test, Pearson’s correlation, and multiple regression test.

**Results:**

The data show that in obese non-diabetic/T2D individuals, TLR8 gene expression was significantly upregulated as compared with lean individuals which correlated with body mass index (BMI) and body fat percentage in non-diabetic population (*P* < 0.05). As expected, TLR8 adipose tissue protein expression in non-diabetic/T2D obese individuals was also higher than that of overweight/lean counterparts. In non-diabetic/T2D individuals, TLR8 gene expression associated (*P* < 0.05) with the expression of CD68, CD11c, CD86, and CD163 macrophage markers. Also, in these individuals, TLR8 gene expression correlated positively (*P* < 0.05) with adipose tissue expression of TNF-α, IL-18, and IL-8 as well as with systemic CRP levels (in non-diabetics). TLR8 expression was also associated with TLR4/TLR2 and MyD88 expression in the adipose tissue.

**Conclusions:**

The elevated adipose tissue expression of TLR8 in obesity/T2D has consensus with inflammatory signatures and may thus represent an immune marker of metabolic inflammation.

**Electronic supplementary material:**

The online version of this article (doi:10.1186/s12950-016-0147-y) contains supplementary material, which is available to authorized users.

## Background

The increasing use of energy-dense foods and overnutrition in recent decades has caused the obesity epidemic to escalate to staggering proportions both in young and adult populations worldwide [1]. Obesity is marked by a state of chronic low-grade inflammation, called metabolic inflammation, in which circulatory monocytes infiltrate the expanding adipose tissue and are differentiated into the adipose tissue macrophages (ATMs). Macrophages in lean mice and humans constitute about 5% of the cells in adipose tissue whereas during obesity, they constitute up to 50% of all adipose tissue cells [2]. The increase in macrophage numbers is accompanied by macrophage activation and expression of proinflammatory cytokines/chemokines that act by autocrine/paracrine mechanisms and may induce insulin resistance in the peripheral tissues [3]. Tumor necrosis factor (TNF)-α, IL-18, IL-8, and C-reactive protein (CRP) are considered potential risk factors for the development of type-2 diabetes (T2D) and its associated metabolic complications [4, 5].

The pathogen- and nutrient-sensing systems are evolutionarily conserved in most species and hence the immunometabolic regulation remains strongly integrated. Toll-like receptors (TLRs) play a key role in the initiation of innate immune responses are regarded as the pattern recognition receptors that recognize pathogen-associated molecular patterns expressed by microbial pathogens or danger-associated molecular patterns expressed by cells during conditions like cellular stress or viral infection [6]. The emerging evidence points to the role of TLRs in non-infectious inflammatory conditions such as rheumatoid arthritis, inflammatory bowel disease, asthma, cancer, and obesity [7–13]. TLR signaling, except TLR3, activates myeloid differentiation factor (MyD)-88 adapter protein which eventually leads to nuclear factor (NF)-κB activation after the macromolecular complex formation including IL1R-associated kinase (IRAK)-1, IRAK-4, tumor necrosis factor-associated factor (TRAF)-6, and IκB kinase complex [14].

TLR8 is associated with sensing of nucleic acids including single-stranded RNA and short double-stranded RNA that are released within the endosomal compartments or by agonists like imidazoquinolines as shown in a mouse model study [15]. Since the TLR8 stimulation was linked to induction of proinflammatory cytokines (TNF-α, IL-1β, IL-6, and IL-12) in peripheral blood mononuclear cells (PBMC), dendritic cells (DCs), and monocytes [16] as well as related with anti-inflammatory IL-10 production [17], we wanted to know if the TLR8 expression was modulated in metabolic disease as has been observed for surface TLRs especially, TLR2 and TLR4. Notably, the changes in the adipose tissue expression of TLR8 in obesity/T2D are not well understood. Herein, we present the data showing increased TLR8 expression in the adipose tissue of obese individuals with or without T2D. We further show that the increased TLR8 expression was concordant with macrophage markers as well as with other inflammatory signatures in the adipose tissue.

## Methods

### Study population

A total of 49 non-diabetic (27 male and 22 female, aged 25–71 years) and 45 T2D individuals (26 male and 19 female, aged 23–72 years) were recruited in the study through clinics of Dasman Diabetes Institute (DDI), Kuwait. All participants gave written informed consent and the study was approved by DDI ethics committee. Those of age <18 years or with serious lung, kidney, liver, or cardiovascular disease, or the ones with hematologic or immune disorders, pregnancy, malignancy or type-1 diabetes were excluded. The participants were sub-classified based on their body mass index (BMI). The non-diabetic group comprised of nine lean (BMI = 22.47 ± 2.52 kg/m^2^; 3 male/6 female), 17 overweight (BMI = 28.36 ± 1.10 kg/m^2^; 11 male/6 female), and 23 obese (BMI = 35.00 ± 3.45 kg/m^2^; 13 male/10 female) individuals. The T2D group comprised of 3 lean (BMI = 25.47 ± 0.34 kg/m^2^; 2 male/1 female), 10 overweight (BMI = 27.87 ± 1.70 kg/m^2^; 6 male/4 female), and 32 obese (BMI = 33.69 ± 2.55 kg/m^2^; 18 male/14 female) individuals. The comorbidities in non-diabetic individuals included milder, early-stage clinical symptoms of hypertension (6), hyperlipidemia (1), coronary artery disease (1), allergy (1), and asthma (2). Similarly, the comorbidities in T2D patients included minor forms of hypertension (18), hyperlipidemia (6), coronary artery disease (2), allergy (2), and asthma (3). The clinico-demographic data of the study participants are summarized in Table 1.

**Table 1:** Patients’ characteristics and clinical data (Groups with significant differences are marked by an asterisk; one-way ANOVA)

Parameter	Non-diabetic			Diabetic		
	Lean	Overweight	Obese	Lean	Overweight	Obese
Total number (N)	9	17	23	3	10	32
Male (N)	3	11	13	2	6	18
Female (N)	6	6	10	1	4	14
Age (Yrs.)	25–53	29–71	26–66	48–58	45–59	23–72
Body mass index (kg/m^2^)	22.47 ± 2.52*	28.36 ± 1.10*	35.00 ± 3.45*	25.47 ± 0.34	27.87 ± 1.70	33.69 ± 2.55
Body fat percentage	27.73 ± 6.09	33.04 ± 4.95	39.09 ± 4.29	32.10 ± 6.22	31.29 ± 6.08	37.33 ± 5.04
Glucose (mmol/L)	4.94 ± 0.72	5.69 ± 1.63	5.24 ± 0.71	5.80 ± 0.42	9.04 ± 2.22	8.71 ± 2.91
Cholesterol (mmol/L)	5.53 ± 1.04	4.87 ± 0.81	5.05 ± 1.14	5.40 ± 2.55	4.91 ± 1.62	5.03 ± 1.23
High-density lipoprotein (mmol/L)	1.70 ± 0.48	1.20 ± 0.19	1.13 ± 0.24	1.14 ± 0.15	1.12 ± 0.40	1.15 ± 0.30
Low-density lipoprotein (mmol/L)	3.53 ± 0.92	3.14 ± 0.74	3.29 ± 1.01	3.70 ± 2.12	2.89 ± 1.37	2.98 ± 1.10
Triglycerides (mmol/L)	0.64 ± 0.25	1.18 ± 0.67	1.44 ± 0.89	1.25 ± 1.25	1.97 ± 0.83	1.89 ± 1.48
HbA1c (%)	5.74 ± 0.49	5.92 ± 1.70	5.68 ± 0.64	6.00 ± 0.28	7.91 ± 1.96	8.27 ± 1.50
Hypertension (N)	0	2	4	0	3	15
Hyperlipidemia (N)	0	0	1	0	2	4
Coronary artery disease (N)	0	0	1	0	1	1
Allergy (N)	0	0	1	0	0	2
Asthma (N)	1	1	0	0	0	3
Therapy	Ventolin	Nadolol, Zocor, Lipitor, Aspirin	Concor, Lipitor, Aspirin, Diovan, Zyloric	Metfornin, Aspirin	Glucophage, Lipitor, Lantus, Zocor, Diovan, NovoRapid, Metformin, Aspirin	NovoRapid, Lantus, Insulin, Aspirin, Concor, Zocor, Mixtard, Tenormin, Glucophage, Metformin, Capoten

### Anthropometric and physioclinical measurements

Height and weight were measured using calibrated portable electronic weighing scales and portable inflexible height measuring bars; the waist circumference was measured using constant tension tape. The whole body composition including body fat percentage, soft lean mass, and total body water were measured using IOI353 Body Composition Analyzer (Jawon Medical, South Korea). Blood pressure was measured by using Omron HEM-907XL digital automatic sphygmomanometer (Omron Healthcare Inc. IL, USA). BMI was calculated as follows: BMI = body weight (kg)/height (m^2^). Peripheral blood was collected from overnight-fasted individuals and analyzed for fasting glucose, glycated hemoglobin (HbA1c), fasting insulin, and lipid profile. Glucose and lipid profiles were measured using Siemens dimension RXL chemistry analyzer (Diamond Diagnostics, Holliston, MA, USA). Glycated hemoglobin (HbA1c) was measured by using Variant™ device (BioRad, Hercules, CA, USA). Plasma high sensitivity CRP (hsCRP) levels were measured using ELISA kit (Biovendor, USA). Plasma triglycerides were also measured using commercial kit (Chema Diagnostica, Monsano, Italy). All assays were carried out following instructions as recommended by the manufacturers.

### Sample collection

Human adipose tissue samples (~0.5 g) were collected via abdominal subcutaneous fat pad biopsy lateral to the umbilicus using standard surgical method. The biopsy tissue was further incised into smaller pieces, rinsed in cold PBS, fixed in 4% paraformaldehyde for 24 h and then embedded in paraffin for further use. Adipose tissue samples (~50-100 mg) were also stored at −80 °C in RNAlater until use.

### Real-time RT-PCR

Total cellular RNA was purified using RNeasy kit (Qiagen, Valencia, CA., USA) and following the manufacturer’s instructions. Samples (1 μg each) were reverse transcribed into cDNA as instructed (High Capacity cDNA Reverse Transcription kit; Applied Biosystems, CA, USA). To perform real-time RT-PCR, cDNA samples (50 ng each) were amplified (40 cycles) using TaqMan® Gene Expression MasterMix (Applied Biosystems, CA, USA) and gene-specific 20× TaqMan gene expression assays as follows: (TLR8) Hs00152972_m1; (CD68) Hs02836816_g1; (CD11c) Hs00174217_m1; (CD86) Hs01567026_m1; (CD163) Hs00174705_m1; (TNF-α) Hs01113624_g1; (IL-18) Hs01038788_m1; (IL-8) Hs00174103_m1; (TLR2) Hs01872448_s1; (TLR4) Hs00152939_m1; (MyD88) Hs01573837_g1; and (GAPDH) Hs03929097_g1 (Applied Biosystems, CA, USA) containing forward and reverse primers and target-specific TaqMan® MGB probe labeled with FAM dye at the 5’ end and NFQ-MGB at the 3’ end of the probe using 7500 Fast Real-Time PCR System (Applied Biosystems, CA, USA). Each cycle involved denaturation (15 s at 95 °C), annealing/extension (1 min at 60 °C) after UDG (2 min at 50 °C) and AmpliTaq gold enzyme (10 min at 95 °C) activation. The amplified GAPDH expression was used as internal control to normalize individual sample differences. TLR8 gene expression level relative to control (lean adipose tissue) was calculated using 2^-ΔΔCt^ method and the relative mRNA expression was expressed as fold expression over the average control gene expression taken as one.

### Immunohistochemistry

Paraffin-embedded adipose tissue sections (4 μm) were deparaffinized in xylene and rehydrated through descending grades (100, 95 and 75%) of ethanol to water. Antigen was retrieved from samples by boiling in retrieval solution (pH6.0; Dako, Glostrup, Denmark) for 8 min in pressure cooker followed by cooling for 15 min. After PBS washing and blocking in 3% H_2_O_2_ for 30 min, 5% nonfat milk for 1 h, and 1% BSA solution for 1 h, samples were treated overnight at room temperature with primary antibody (1:800 diluted anti-human TLR8 mouse monoclonal antibody; Abcam® ab85859). After two washes with PBS-0.5% Tween, samples were treated for 1 h with HRP-conjugated goat anti-mouse secondary antibody (EnVision™ Kit, Dako, Glostrup, Denmark) and color was developed using 3,3’-diaminobenzidine (DAB) substrate. Specimens were washed in running tap water, counterstained with Harris hematoxylin, dehydrated through ascending grades (75, 95, and 100%) of ethanol, cleared in xylene, and mounted in dibutyl phthalate xylene (DPX). For quantitative analysis of TLR8 protein expression, the entire adipose tissue sections (100×; Panoramic Scan, 3D-HISTECH, Hungary) were used to quantify immunohistochemical staining in all subdivided sample regions that were outlined using Aperio ImageScope software (Aperio Vista, CA, USA). On average, 700 cells per sample were counted excluding the connective tissue and blood vessels. Aperio-positive pixel count algorithm (version 9) was used for quantitative analysis of TLR8 immunostaining in the regions sampled. The number of positive pixels was normalized to the number of total (positive and negative) pixels to account for variations in the size of the region sampled. Color and intensity thresholds were established to detect the specific immunostaining as positive and the background staining as negative pixels. Once the conditions were set, all slides were analyzed using same parameters.

### Confocal microscopy

Formalin-fixed paraffin-embedded adipose tissue sections (8 μm) were immunolabeled as described before. Following antigen retrieval and blocking, samples were treated overnight at room temperature with primary antibody (1:100 diluted anti-TLR8 monoclonal antibody; abcam® ab85859). After two washes with PBS-0.05% Tween, samples were incubated for 1 h with secondary antibody (1:200 diluted goat anti-mouse Alexa Fluor® 647-conjugated antibody; Abcam® ab150115) and washed three times in PBS. Samples were counterstained with 4’,6-diamidino- 2-phenylindole (DAPI) (Vectashield, Vector Laboratories, H-1500) and mounted. For image processing and analysis, confocal images were collected (Inverted Zeiss LSM710 spectral confocal microscope, Carl Zeiss, Gottingen, Germany) using EC Plan-Neofluar 40×/1.30 oil DIC M27 objective lens. Samples were excited using a 488 nm diode-pumped solid-state laser and the 405 nm line of an argon ion laser. After laser excitation, optimized emission detection bandwidths were configured using Zeiss Zen 2010 control software.

### Statistical analysis

Data were expressed as mean ± SEM values unless otherwise indicated and statistical analysis was performed using GraphPad Prism software (La Jolla, CA, USA) and SPSS for Windows version 19.01 (IBM SPSS Inc. USA). Unpaired Student *t*-test and Mann-Whitney *U*-test were used to compare group means. Correlation and stepwise multiple regression were performed to determine associations between different variables. One-way ANOVA was used to identify groups that had significant differences. All *P*-values <0.05 were considered significant.

## Results

### Increased adipose tissue TLR8 gene expression in diabetic/non-diabetic obese individuals

**Fig. 1:**
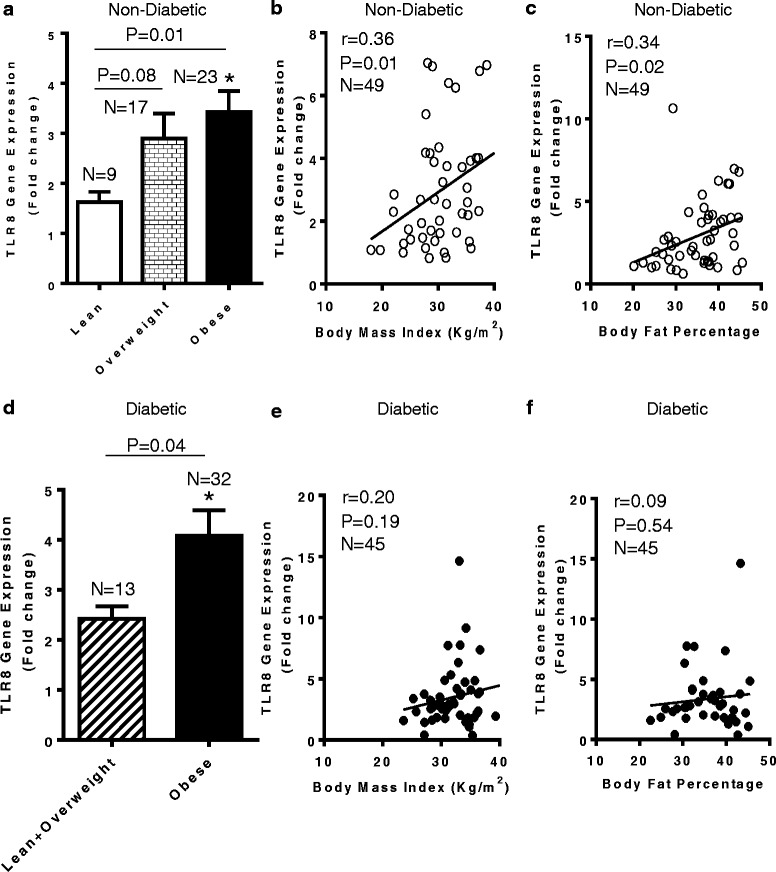
Upregulated TLR8 gene expression in the adipose tissue of obese individuals with or without type-2 diabetes. TLR8 gene expression in the subcutaneous adipose tissue biopsy samples collected from 49 non-diabetic (23 obese, 17 overweight, and nine lean) and 45 type-2 diabetic (T2D) (32 obese, ten overweight, and three lean) individuals was determined by quantitative real-time RT-PCR as described in Methods. The representative data from three independent determinations are shown. (**a**) TLR8 mRNA expression in non-diabetic individuals was found to be significantly upregulated in the adipose tissue samples of obese as compared with lean individuals (*P* = 0.01). The increased TLR8 gene expression correlated positively with (**b**) body mass index (BMI) (*r* = 0.36; *P* = 0.01) and (**c**) body fat percentage (*r* = 0.34; *P* = 0.02). (**d**) TLR8 mRNA expression in T2D patients was also found to be significantly upregulated in the adipose tissue samples from obese as compared with lean and overweight subjects combined (*P* = 0.04). However, in this cohort, TLR8 gene expression did not correlate with (**e**) BMI (*r* = 0.20; *P* = 0.19) and (**f**) body fat percentage (*r* = 0.09; *P* = 0.54)

Whereas the changes in TLR2/TLR4 expression in the adipose tissue are regarded as important actors in metabolic inflammation, the changes in the adipose tissue expression of endocytic TLR8 in obesity/T2D remain unclear. To this end, we found that in non-diabetic individuals, TLR8 mRNA expression in the adipose tissue was significantly upregulated in obese as compared with lean counterparts (*P* = 0.01) (Fig. 1a) and this increase correlated positively with phenotypes of corpulence, such as BMI (*r* = 0.36, *P* = 0.01) (Fig. 1b) and body fat percentage (*r* = 0.34, *P* = 0.02) (Fig. 1c). The adipose tissue TLR8 mRNA expression was also found to be higher in obese T2D patients as compared with lean/overweight population (*P* = 0.04) (Fig. 1d). The expression of TLR8 in the diabetic cohort did not associate with parameters of corpulence including BMI (*r* = 0.19, *P* = 0.19) (Fig. 1e) and body fat percentage (*r* = 0.09, *P* = 0.54) (Fig. 1f) and, instead, it showed an association with the metabolic parameters including glycemia and glycated hemoglobin (HbA1c) levels. (Additional file 1).

### Elevated adipose tissue TLR8 protein expression in obesity

**Fig. 2:**
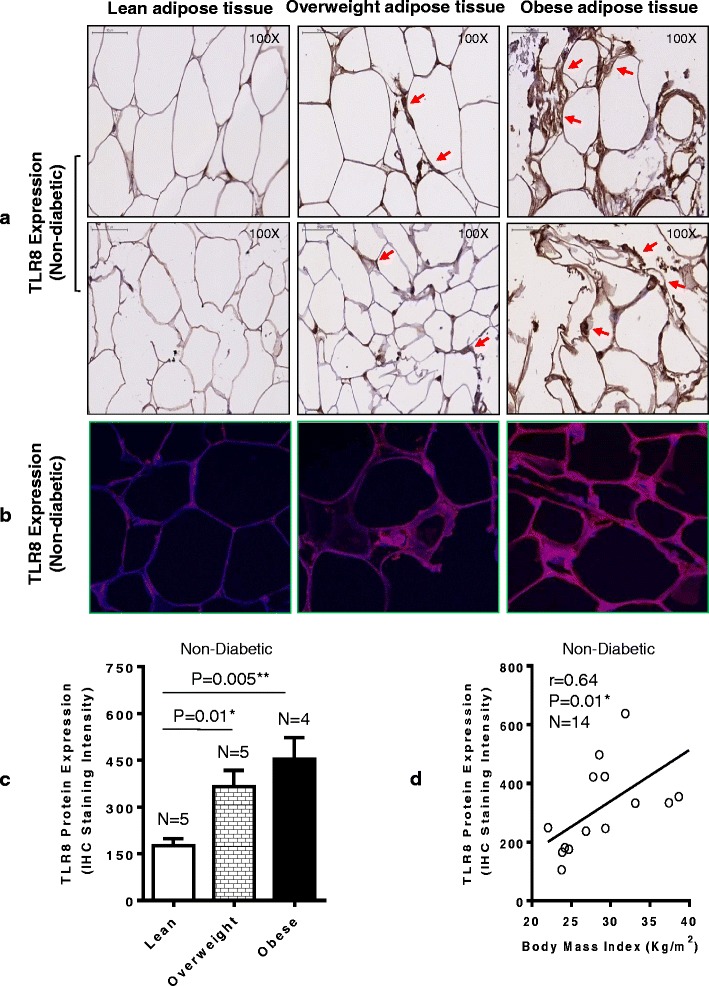
Elevated TLR8 protein expression in the adipose tissue samples from obese and overweight non-diabetic individuals. TLR8 protein expression in the subcutaneous adipose tissue samples from 15 non-diabetic individuals comprising lean, overweight and obese, five each, was detected by immunohistochemistry (IHC) and confirmed by confocal microscopy as described in Methods. TLR8 protein expression in the adipose tissue samples was quantified using Aperio ImageScope software (Aperio Vista, CA, USA) and algorithm (version 9); on average, 700 cells were counted for each sample. The representative data from three independent determinations are shown. TLR8 protein expression is shown by using (**a**) immunohistochemistry (*arrows*); and (**b**) confocal microscopy where *red* color represents TLR8-specific staining and *blue* color represents nuclei staining (40× magnification). **c** TLR8 protein expression quantified as IHC staining intensity shows significantly higher expression in obese (*P* = 0.005) and overweight (*P* = 0.01) individuals as compared with lean counterparts. (**d**) The adipose tissue TLR8 protein expression in non-diabetics correlates with BMI (*r* = 0.64 *P* = 0.01)

**Fig. 3:**
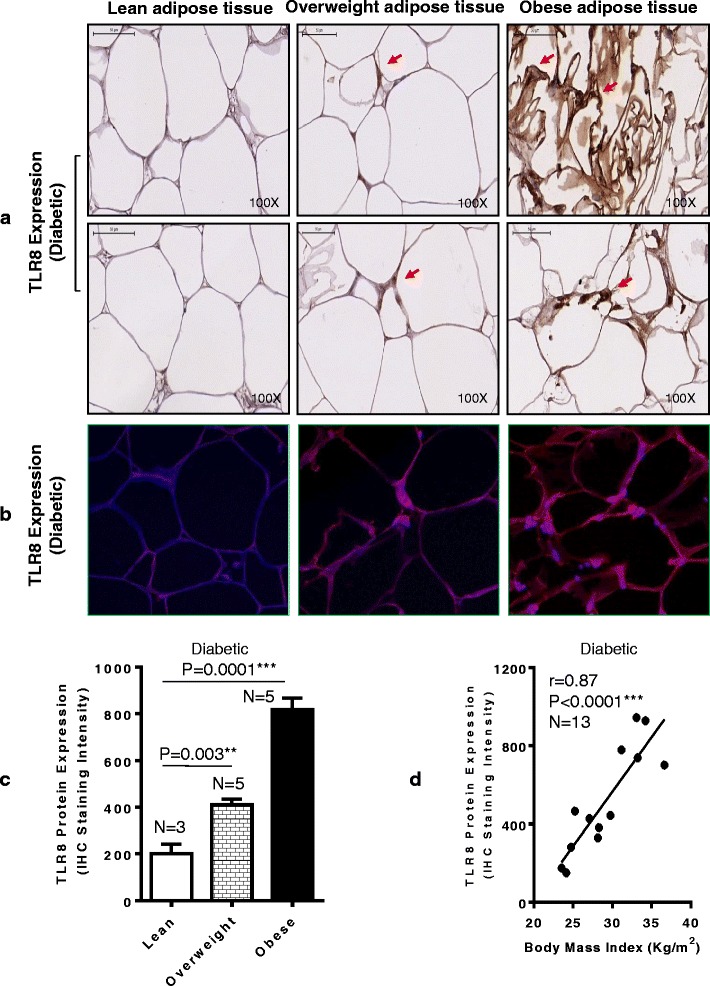
Increased TLR8 protein expression in the adipose tissue samples from obese and overweight type-2 diabetic (T2D) individuals. TLR8 protein expression in the subcutaneous adipose tissue samples from 13 T2D patients comprising three lean, five overweight and five obese individuals was detected by immunohistochemistry (IHC) and confirmed by confocal microscopy as described in Methods. TLR8 protein expression in the adipose tissue samples was quantified by using Aperio ImageScope software (Aperio Vista, CA, USA) and algorithm (version 9); while on average, 700 cells were counted for each sample. The representative data from three independent determinations are shown. TLR8 protein expression is shown by using (**a**) immunohistochemistry (*arrows*); (**b**) confocal microscopy wherein *red* color represents TLR8-specific staining and *blue* color represents nuclei staining (40× magnification). (**c**) TLR8 protein expression quantified as IHC staining intensity shows significantly higher expression in obese (*P* = 0.0001) and overweight (*P* = 0.003) individuals as compared with lean counterparts. (**d**) The adipose tissue TLR8 protein expression in T2D patients correlates with BMI (*r* = 0.87 *P* < 0.0001)

We further asked whether the TLR8 protein expression was also elevated in the adipose tissue in obesity. As expected, we found that the adipose tissue TLR8 protein expression in 14 non-diabetic individuals as analyzed by immunohistochemistry (shown for two individuals per group; Fig. 2a) and confirmed by confocal microscopy (shown for one individual in each group; Fig. 2b) was significantly elevated in both obese (*P* = 0.005) and overweight (*P* = 0.01) subjects as compared with lean controls (Fig. 2c). The elevated TLR8 protein expression in this cohort was also found to correlate with BMI (*r* = 0.64, *P* = 0.01) (Fig. 2d). Similarly in 13 T2D individuals, the adipose tissue TLR8 protein expression determined by immunohistochemistry (shown for two individuals per group; Fig. 3a) and confirmed by confocal microscopy (shown for one individual in each group; Fig. 3b) was found to be elevated in obese (*P* = 0.0001) and overweight (*P* = 0.003) as compared with lean counterparts (Fig. 3c); and TLR8 protein expression associated with BMI in diabetic cohort (*r* = 0.87, *P* < 0.0001) (Fig. 3d). As shown in Additional file 2, overall, a highly significant positive correlation was found between gene and protein expression of TLR8 (*r* = 0.86, *P* < 0.0001).

### TLR8 gene expression correlates with monocyte/macrophage markers in the adipose tissue

**Fig. 4:**
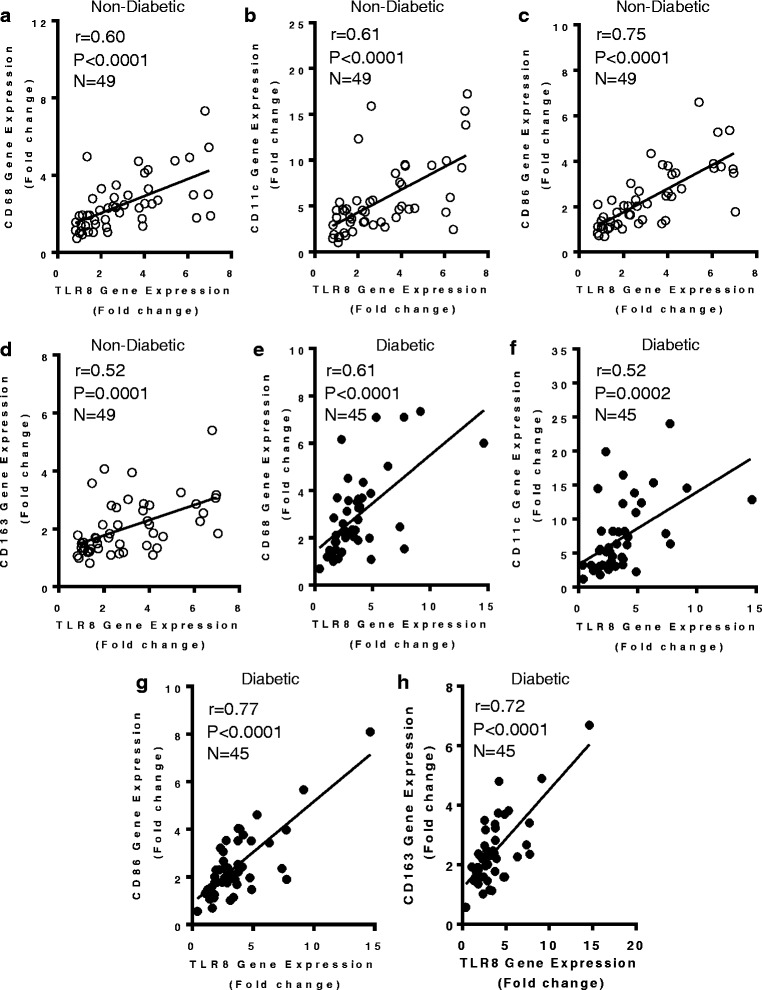
TLR8 gene expression in the adipose tissue of non-diabetic/diabetic individuals correlates with monocyte/macrophage markers. The adipose tissue gene expression of TLR8 and monocyte/macrophage markers (CD68, CD11c, CD86, and CD163) in 49 non-diabetic (ND) and 45 type-2 diabetic (T2D) individuals was determined by using quantitative real-time RT-PCR as described in Methods. The representative data from three independent determinations show that TLR8 gene expression correlated positively/significantly with the local expression of (**a**) CD68_[ND]_ (*r* = 0.60, *P* < 0.0001); (**b**) CD11c_[ND]_ (*r* = 0.61, *P* < 0.0001); (**c**) CD86_[ND]_ (*r* = 0.75, *P* < 0.0001); (**d**) CD163_[ND]_ (*r* = 0.52, *P* = 0.0001); (**e**) CD68_[T2D]_ (*r* = 0.61, *P* < 0.0001); (**f**) CD11c_[T2D]_ (*r* = 0.52, *P* = 0.0002); (**g**) CD86_[T2D]_ (*r* = 0.77, *P* < 0.0001); and (**h**) CD163_[T2D]_ (*r* = 0.72, *P* < 0.0001)

We next asked if the increased TLR8 mRNA expression in the adipose tissue was concordant with a local inflammatory profile marked by an increased expression of monocyte/macrophage markers. In this regard, we found that in non-diabetic individuals, the adipose tissue TLR8 gene expression correlated positively with the gene expression of CD68 (*r* = 0.60, *P* < 0.0001) (Fig. 4a), CD11c (*r* = 0.61, *P* < 0.0001) (Fig. 4b), CD86 (*r* = 0.75, *P* < 0.0001) (Fig. 4c), and CD163 (*r* = 0.52, *P* = 0.0001) (Fig. 4d). Also in T2D patients, TLR8 gene expression correlated positively with CD68 (*r* = 0.61, *P* < 0.0001) (Fig. 4e), CD11c (*r* = 0.52, *P* = 0.0002) (Fig. 4f), CD86 (*r* = 0.77, *P* < 0.0001) (Fig. 4g), and CD163 (*r* = 0.72, *P* < 0.0001) (Fig. 4h). While analyzing in a total population of 94 individuals (49 non-diabetic and 45 diabetic), the adipose tissue expression of macrophage markers CD68, CD86, and CD163 associated positively with BMI (*P* < 0.05) (Additional file 3); however, CD11c did not associate with BMI (*r* = 0.12, *P* = 0.21) (data not shown).

### Increased TLR8 gene expression in obese adipose tissue associates with an inflammatory profile

**Fig. 5:**
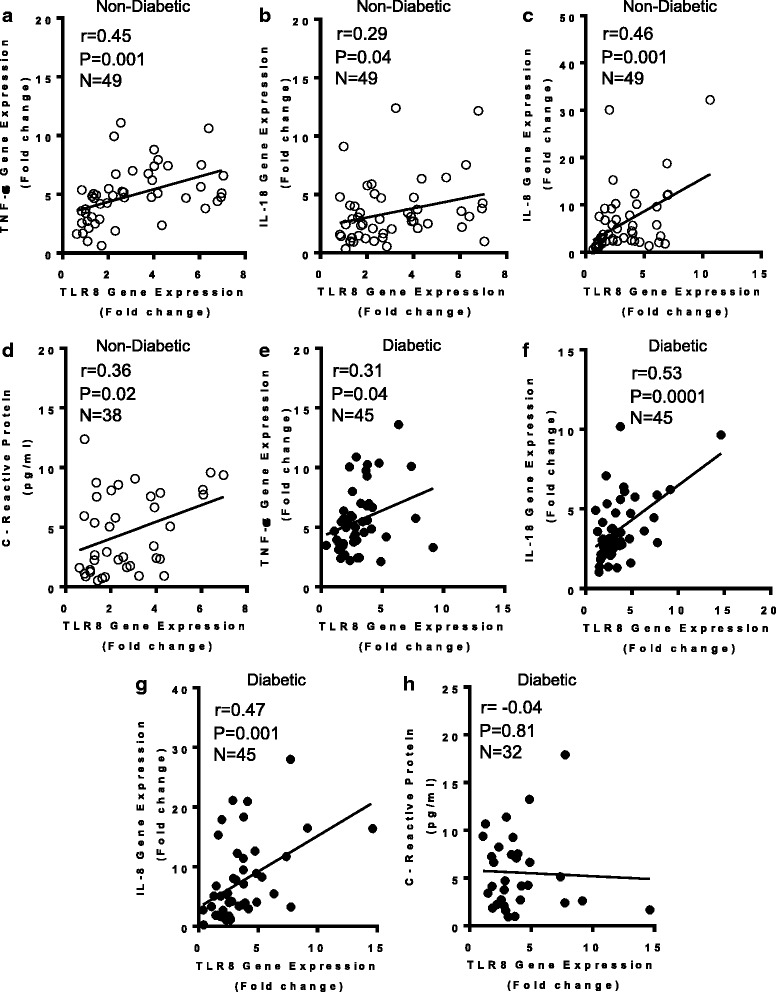
Increased adipose tissue TLR8 gene expression correlates with typical inflammatory markers. The adipose tissue gene expression of TLR8 and signature inflammatory cytokines/chemokines (TNF-α, IL-18, and IL-8) was determined in 49 non-diabetic (ND) and 45 type-2 diabetic (T2D) individuals using quantitative real-time RT-PCR while plasma high-sensitivity C-reactive protein (CRP) levels were determined using commercial ELISA kit and following the manufacturer’s recommendations. The representative data from two independent determinations show that TLR8 mRNA expression was associated with the expression of (**a**) TNF-α_[ND]_ (*r* = 0.45, *P* = 0.001); (**b**) IL-18_[ND]_ (*r* = 0.29, *P* = 0.04); (**c**) IL-8_[ND]_ (*r* = 0.46, *P* = 0.001); (**d**) Plasma CRP_[ND]_ (*r* = 0.36, *P* = 0.02); (**e**) TNF-α_[T2D]_ (*r* = 0.31, *P* = 0.04); (**f**) IL-18_[T2D]_ (*r* = 0.53, *P* = 0.0001); and (**g**) IL-8_[T2D]_ (*r* = 0.47, *P* = 0.001). (**h**) TLR8 gene expression in T2D patients did not correlate with plasma CRP levels (*r* = −0.04, *P* = 0.81). Due to missing plasma samples, TLR8 correlation with plasma CRP levels is shown for 38 ND and 32 T2D individuals

We further wanted to know if the elevated TLR-8 gene expression in obese adipose tissues was in agreement with the increased expression of inflammatory cytokines/chemokines and related markers. To this end, we found that in non-diabetic persons, the adipose tissue TLR8 mRNA expression associated positively with gene expression of TNF-α (*r* = 0.45, *P* = 0.001) (Fig. 5a), IL-18 (*r* = 0.29, *P* = 0.04) (Fig. 5b), IL-8 (*r* = 0.46, *P* = 0.001) (Fig. 5c), and plasma CRP levels (*r* = 0.36, *P* = 0.02) (Fig. 5d). In T2D patients, TLR8 gene expression correlated with TNF-α (*r* = 0.31, *P* = 0.04) (Fig. 5e), IL-18 (*r* = 0.53, *P* = 0.0001) (Fig. 5f), and IL-8 (*r* = 0.47, *P* = 0.001) (Fig. 5g); whereas, no correlation was found with plasma CRP levels (*r* = −0.04, *P* = 0.81) (Fig. 5h).

### TLR8 expression in the adipose tissue is linked with TLR2/TLR4 and MyD88 expression

**Fig. 6:**
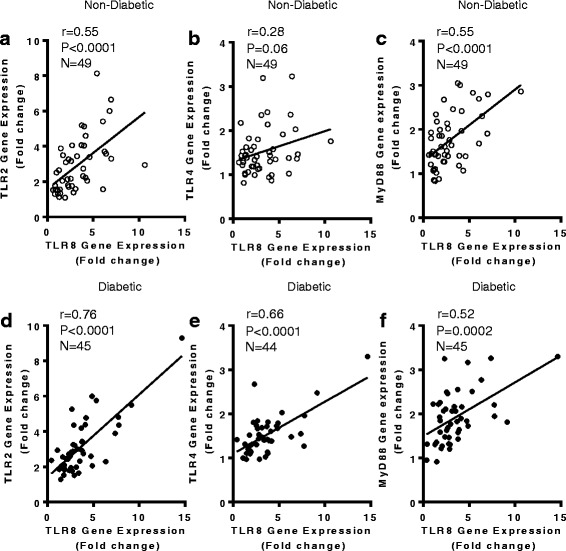
TLR8 mRNA expression in the adipose tissue associates with local gene expression of TLR2, TLR4, and MyD88. The adipose tissue gene expression of TLR2, TLR4, TLR8, and MyD88 was determined in 49 non-diabetic and 45 type-2 diabetic (T2D) individuals by using quantitative real-time RT-PCR as described in Methods. The representative data from two independent determinations show that TLR8 gene expression was positively associated with that of (**a**) TLR2 (*r* = 0.55, *P* < 0.0001); (**b**) TLR4 (*r* = 0.28, approaching significance with *P* = 0.06); and (**c**) MyD88 (*r* = 0.55, *P* < 0.0001). Similarly, in T2D patients, TLR8 gene expression was found to be associated with (**d**) TLR2 (*r* = 0.76, *P* < 0.0001); (**e**) TLR4 (*r* = 0.66, *P* < 0.0001); and (**f**) MyD88 expression (*r* = 0.52, *P* = 0.0002). Due to missing data, TLR8 correlation with TLR4 is shown for 44 T2D patients

The innate immune TLRs have potential to play direct or indirect roles in obesity- or T2D-associated metabolic inflammation and, therefore, we asked whether the adipose tissue TLR8 gene expression was linked with the expression of other immunometabolic TLRs such as TR2 and TLR4 as well as with downstream MyD88 adapter protein. To this effect, we found that TLR8 gene expression in non-diabetic individuals was positively associated with the adipose tissue gene expression of TLR2 (*r* = 0.55, *P* < 0.0001) (Fig. 6a), TLR4 (*r* = 0.28, approaching the level of statistical significance with *P* = 0.06) (Fig. 6b), and MyD88 (*r* = 0.55, *P* < 0.0001) (Fig. 6c). Similarly, in T2D patients as well, TLR8 gene expression was associated with that of TLR2 (*r* = 0.76, *P* < 0.0001) (Fig. 6d), TLR4 (*r* = 0.66, *P* < 0.0001) (Fig. 6e), and MyD88 expression (*r* = 0.52, *P* = 0.0002) (Fig. 6f).

Additionally, within-group correlations of the TLR8 gene expression with other inflammatory markers in the adipose tissue were also determined and the data are summarized in Additional file 4.

## Discussion

The emerging evidence supports that immune and metabolic systems are tightly integrated and the innate immune TLRs can be directly or indirectly activated by overnutrition leading to metabolic inflammation. Regarding the adipose tissue changes in TLR8 expression in obesity/T2D, our data show that in non-diabetic obese individuals, the upregulated TLR8 mRNA expression correlated with corpulence-related phenotypes including BMI and body fat percentage. However, the altered TLR8 expression did not associate with corpulence phenotypes in T2D individuals that happen to be more heterogeneous due to presence of dyslipidemia and related complications. Instead, TLR8 gene expression in this cohort was associated with metabolic traits e.g. glycemia and HbA1c levels. It is not clear whether hyperglycemia could upregulate TLR8 expression, and a few studies both in humans and mice have shown the link between hyperglycemia and TLR2/4 induction and upregulation [18, 19]. Our data also show elevated TLR8 protein expression in the adipose tissue in obesity with/without T2D and, notably, a good agreement (*r* = 0.86, *P* < 0.0001) was found between gene and protein expression of TLR8. Although, the global expression of TLR8 detected in the adipose tissue may relate to a variety of cells present in this compartment, it is mainly expressed by monocytes/macrophages, mast cells, and myeloid dendritic cells [20]. The increased TLR8 expression detected by immunohistochemical staining was found on inflammatory cell aggregates that were visible as crown like structures (CLS) surrounding the degenerating adipocytes in obese adipose tissue samples (arrow heads point to CLS).

Next, we found that the increased TLR8 gene expression in the adipose tissues in obesity/T2D paralleled with enhanced expression of monocyte/macrophage markers such as CD68, CD11c, CD86, and CD163. The CD68, CD11c, and CD86 are markers of inflammatory or M1-type macrophages and CD163 is a hemoglobin-haptoglobin scavenger receptor. We found that the adipose tissue expression of these markers was associated with corpulence phenotype i.e. BMI which meant that obesity could be a positive modulator of monocyte/macrophage influx into the adipose tissue which is known to secrete chemotactic adipokines including CC-chemokine ligand (CCL)-2 or macrophage chemoattractant protein (MCP)-1 as shown earlier by a diet-induced obesity study in mice [21]. Our data showing elevated macrophage markers expression in the adipose tissue in obesity/T2D are corroborated, at least in part, by other studies as well [2, 22, 23]. Of note, Devevre et al. reported three distinct monocytic populations in human obesity named as classical, intermediate, and non-classical monocytes while a 2–4 fold increased expression of TLR4/TLR8 was found in all three subsets [24]. These observations partially support our data showing enhanced TLR8 expression in the obese adipose tissue.

We also found that TLR8 gene expression was positively associated with inflammatory cytokines/chemokine expression in the adipose tissue as well as with circulatory levels of inflammatory biomarker CRP. The white adipose tissue is a site for excessive energy storage and active biosynthesis of adipokines that act via autocrine/paracrine mechanisms in adipocyte-macrophage crosstalk to amplify the local and systemic inflammatory responses in obesity/T2D [25]. The proinflammatory cytokines TNF-α and IL-18 are known to be elevated in obesity/T2D [26, 27] while IL-8 or CXC chemokine ligand (CXCL)-8 is an inflammatory protein and increased circulatory numbers of IL8-expressing monocytes were also reported in human obesity [24]. CRP is a typical systemic inflammatory marker and its increased levels were linked with obesity, blood pressure, and hyperlipidemia [28]. The proinflammatory bioactive proteins TNF-α, IL-18, and IL-8 are intertwined through cross-regulatory mechanisms. TNF-α was found to regulate IL-8 in human adipose tissue [29] and IL-18 in human adipocytes [30] whereas, IL-18 induced IL-8 and IL-1β in human monocytes in a TNFα-dependent mechanism [31]. Our data showing increased TNF-α, IL-18, and IL-8 expression in obesity are in agreement with studies showing induction of these proinflammatory proteins in obese humans [32, 33]. Our data further show an association between these inflammatory adipokines and TLR8 expression in the adipose tissue; however, CRP levels in T2D individuals did not correlate with TLR8 gene expression and we speculate that this discrepancy may be due to T2D-associated comorbid factors such as liver dysfunction, central adiposity, hyperglycemia, and insulin resistance. This line of argument is supported by the observation that studies on plasma CRP levels in T2D patients show heterogeneity in their findings [34, 35]. Also, we cannot rule out plausible effects of anti-diabetic pharmacologic interventions on systemic levels of CRP [36]. Multiple stepwise linear regression analysis of our data revealed that CD11c, CD86, and IL-8 independently predicted TLR8 gene expression in the non-diabetic while CD11c predicted TLR8 gene expression in the diabetic subjects. Additionally, analysis of the within-group associations of TLR8 gene expression with other inflammatory markers in the adipose tissue indicates that obesity- and T2D-associated inflammatory changes including macrophage infiltration, upregulated expression of proinflammatory cytokines/chemokines, increased surface TLRs (TLR2/4) and downstream adapter protein MyD88 may be linked, at different levels, to the induction/upregulation of TLR8 in the adipose tissue.

The altered TLR expression observed in metabolic disease conditions is likely to have pathological consequences. We show that TLR8 gene expression was positively associated with TLR2, TLR4, and MyD88 expression in the adipose tissue. The upregulation of TLRs and inflammatory cytokine production have been also reported by previous studies in humans [13, 37]. TLR2/4 bind to free fatty acids in addition to other known ligands and MyD88 is a downstream adapter protein which is involved in TLR-mediated signaling except TLR3 [38]. While several studies point to a link between TLR upregulation and metabolic inflammation [13, 37, 39], our data add the adipose tissue changes in endocytic TLR8 as an immune marker to the growing list of novel correlates of metabolic inflammation. Human TLR8 is a part of the nucleic acid-sensing TLRs that recognize viral ss/ds RNAs [40] and bacterial RNA [41]; however, TLR8 agonist(s) associated with obesity/T2D are still not known. Therefore, TLR8 expression changes need to be further investigated with regard to putative agonists like cellular RNAs and alarmins (e.g. HMGB1) which are known to be released during lipolysis in morbid obesity [42]. Besides, the present study is also limited by certain factors as follows: (1) small cohort size for each BMI group; (2) the lack of data expressing functional changes in relation to altered TLR8 expression in the adipose tissue; and (3) the data represent TLR8 global changes in the adipose tissue in obesity/T2D and hence selective expression of TLR8 in adipocytes and macrophages remains unknown.

## Conclusions

Taken together, the present data show significantly elevated TLR8 mRNA and protein expression in the adipose tissue in obesity/T2D. Based on consensus with other inflammatory markers, TLR8 expression changes in the obese adipose tissue may represent a novel molecular signature of metabolic inflammation.

## Additional files

Additional file 1: Correlation between TLR8 gene expression in the adipose tissue and glycemia/HbA1c levels. The adipose tissue TLR8 gene expression in 45 type-2 diabetic individuals (32 obese, 10 overweight, and three lean) was determined by quantitative real-time RT-PCR and fasting blood glucose (mmol/L) and glycated hemoglobin (HbA1c) (%) levels were determined using commercial kits as recommended by the manufacturers. The data show that TLR8 gene expression correlated positively with (A) glycemia (*r* = 0.31, *P* = 0.04) and (B) HbA1c levels (*r* = 0.34, *P* = 0.02). (PDF 15 kb)
Additional file 2: Correlation between TLR8 gene and protein expression in the adipose tissue. The adipose tissue TLR8 gene and protein expression was determined in 21 individuals (15 non-diabetic and 6 diabetic) classified as five lean, eight overweight and eight obese by using quantitative real-time RT-PCR and immunohistochemistry, respectively. TLR8 relative mRNA expression was measured as fold change over the average of control gene expression assumed as one. TLR8 protein expression was measured as staining intensity which was quantified using Aperio-positive pixel count algorithm (version 9). The number of positive pixels was normalized to the number of total (positive and negative) pixels to account for variations in the size of the regions sampled. Color and intensity thresholds were established to detect the immunostaining as positive and background staining as negative pixels. The positive correlation between TLR8 gene and protein expression was found to be highly significant (*r* = 0.86, *P* < 0.0001). (PDF 8 kb)
Additional file 3: Correlation between macrophage markers expression in the adipose tissue and body mass index. The adipose tissue gene expression of various monocyte/macrophage markers including CD68, CD86, and CD163 in 94 individuals (49 non-diabetic and 45 type-2 diabetic) was determined by using quantitative real-time RT-PCR as described in Methods. The data show significant positive associations between body mass index (BMI) and adipose tissue gene expression of (A) CD68 (*r* = 0.26, *P* = 0.007), (B) CD86 (*r* = 0.20, *P* = 0.04), and (C) CD163 (*r* = 0.27, *P* = 0.005) macrophage markers. (PDF 115 kb)
Additional file 4: Within-group correlations of TLR8 expression with other inflammatory markers in the adipose tissue. (DOCX 22 kb)

## Abbreviations

ATMs: Adipose tissue macrophages
BMI: Body mass index
CCL-2: CC chemokine ligand-2
CLS: Crown-like structures
CRP: C-reactive protein
CXCL-8: CXC chemokine ligand-8
DAB: 3,3’-Diaminobenzidine
DAPI: 4’,6-Diamidino- 2-phenylindole
DCs: Dendritic cells
DPX: Dibutyl phthalate xylene
HbA1c: Glycated hemoglobin
hsCRP: High-sensitivity CRP
IRAK: IL-1R-associated kinase
MCP-1: Macrophage chemoattractant protein-1
MyD88: Myeloid differentiation factor-88
NFκB: Nuclear factor kappa B
T2D: Type-2 diabetes
TLRs: Toll-like receptors
TNF-α: Tumor necrosis factor-alpha
TRAF: Tumor necrosis factor-associated factor
