# Supplementary figures and images for: Increased adipose tissue expression of TLR8 in obese individuals with or without type-2 diabetes: significance in metabolic inflammation
Source: J Inflamm (Lond). 2016 Dec 8;13:38. doi: 10.1186/s12950-016-0147-y (PMC5146894; doi:10.1186/s12950-016-0147-y)

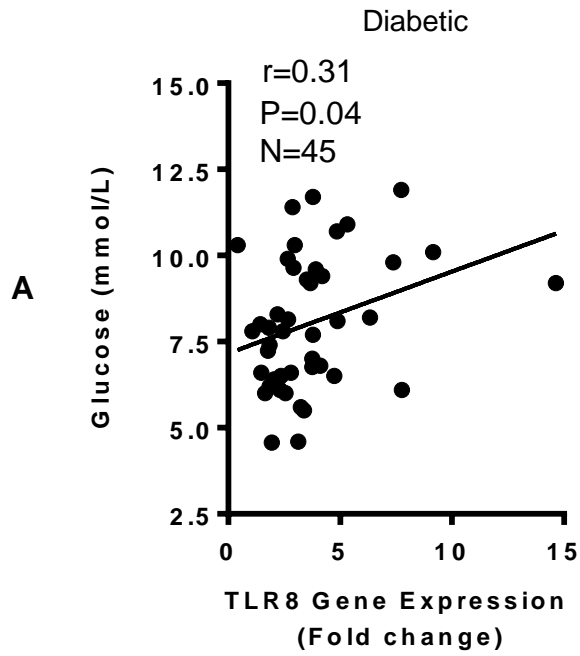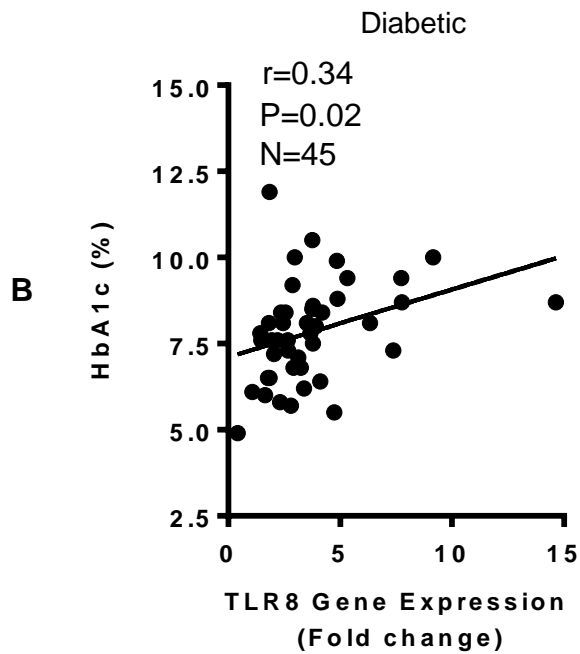

Supplement: Additional file 1: — Correlation between TLR8 gene expression in the adipose tissue and glycemia/HbA1c levels. The adipose tissue TLR8 gene expression in 45 type-2 diabetic individuals (32 obese, 10 overweight, and three lean) was determined by quantitative real-time RT-PCR and fasting blood glucose (mmol/L) and glycated hemoglobin (HbA1c) (%) levels were determined using commercial kits as recommended by the manufacturers. The data show that TLR8 gene expression correlated positively with (A) glycemia (r = 0.31, P = 0.04) and (B) HbA1c levels (r = 0.34, P = 0.02). (PDF 15 kb) [file 12950_2016_147_MOESM1_ESM.pdf]

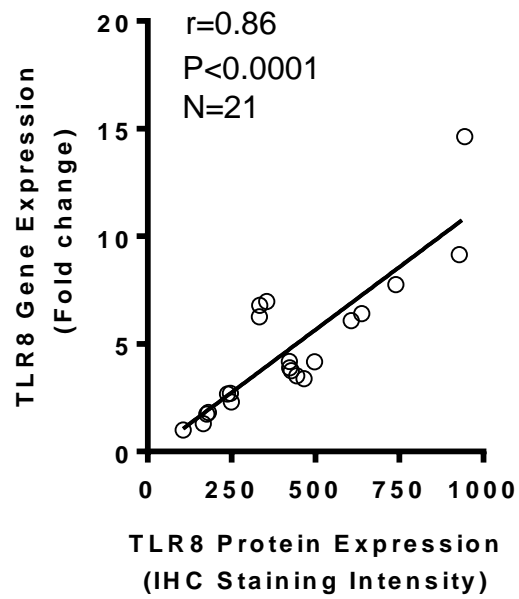

Supplement: Additional file 2: — Correlation between TLR8 gene and protein expression in the adipose tissue. The adipose tissue TLR8 gene and protein expression was determined in 21 individuals (15 non-diabetic and 6 diabetic) classified as five lean, eight overweight and eight obese by using quantitative real-time RT-PCR and immunohistochemistry, respectively. TLR8 relative mRNA expression was measured as fold change over the average of control gene expression assumed as one. TLR8 protein expression was measured as staining intensity which was quantified using Aperio-positive pixel count algorithm (version 9). The number of positive pixels was normalized to the number of total (positive and negative) pixels to account for variations in the size of the regions sampled. Color and intensity thresholds were established to detect the immunostaining as positive and background staining as negative pixels. The positive correlation between TLR8 gene and protein expression was found to be highly significant (r = 0.86, P < 0.0001). (PDF 8 kb) [file 12950_2016_147_MOESM2_ESM.pdf]

**A**

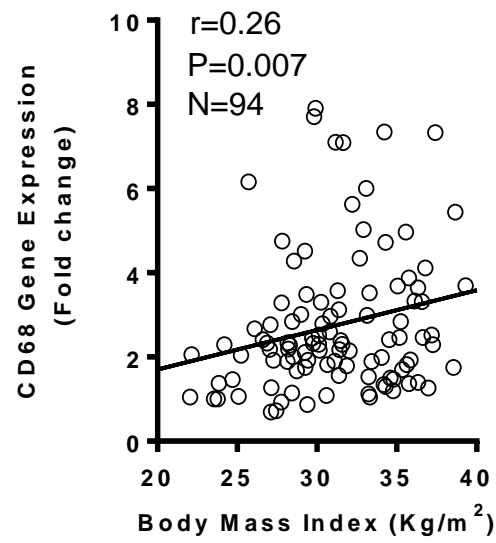

**B**

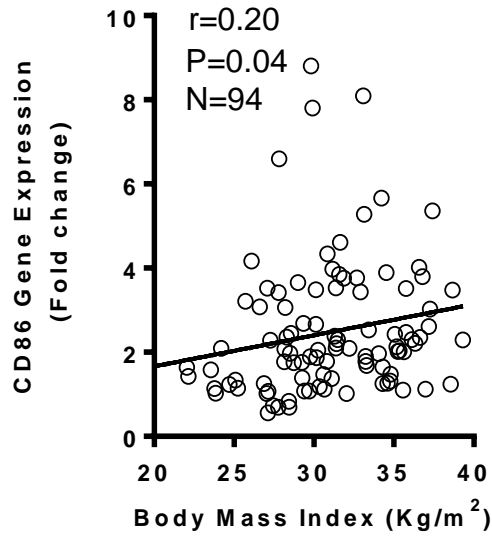

**C**

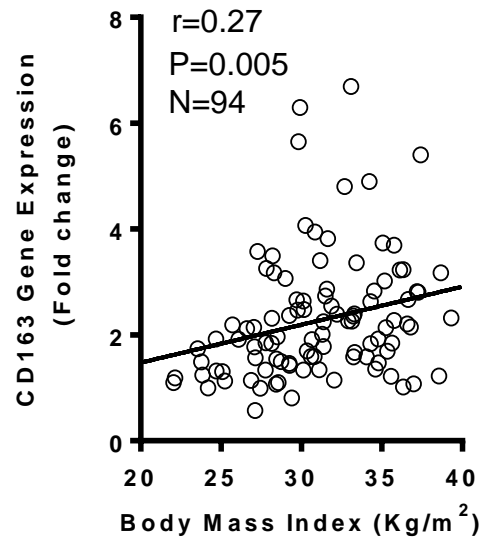

Supplement: Additional file 3: — Correlation between macrophage markers expression in the adipose tissue and body mass index. The adipose tissue gene expression of various monocyte/macrophage markers including CD68, CD86, and CD163 in 94 individuals (49 non-diabetic and 45 type-2 diabetic) was determined by using quantitative real-time RT-PCR as described in Methods. The data show significant positive associations between body mass index (BMI) and adipose tissue gene expression of (A) CD68 (r = 0.26, P = 0.007), (B) CD86 (r = 0.20, P = 0.04), and (C) CD163 (r = 0.27, P = 0.005) macrophage markers. (PDF 115 kb) [file 12950_2016_147_MOESM3_ESM.pdf]
