# Supplementary material for: Increased adipose tissue expression of TLR8 in obese individuals with or without type-2 diabetes: significance in metabolic inflammation
Source: J Inflamm (Lond). 2016 Dec 8;13:38. doi: 10.1186/s12950-016-0147-y (PMC5146894; doi:10.1186/s12950-016-0147-y)
Supplement: Additional file 4: — Within-group correlations of TLR8 expression with other inflammatory markers in the adipose tissue. (DOCX 22 kb) [file 12950_2016_147_MOESM4_ESM.docx]

| **Non-diabetic Obese** | | | **Diabetic Obese** | | |
| --- | --- | --- | --- | --- | --- |
| ***Inflammatory marker*** | ***Pearson (r)*** | ***P-value*** | ***Inflammatory marker*** | ***Pearson (r)*** | ***P-value*** |
| CD86 | 0.80 | <0.0001 | CD86 | 0.77 | 0.0001 |
| CD163 | 0.60 | 0.001 | CD163 | 0.72 | <0.0001 |
| TLR2 | 0.56 | 0.003 | TLR2 | 0.81 | <0.0001 |
| TLR4 | 0.41 | 0.04 | TLR4 | 0.80 | <0.0001 |
| MyD88 | 0.38 | 0.03 | MyD88 | 0.60 | 0.0001 |
|  |  |  | CD68 | 0.59 | 0.002 |
|  |  |  | CD11c | 0.60 | 0.001 |
|  |  |  | CXCL-8/IL-8 | 0.50 | 0.002 |
|  |  |  | CCL-2/MCP-1 | 0.61 | 0.001 |
| **Non-diabetic Overweight** | | | **Diabetic Overweight** | | |
| ***Inflammatory marker*** | ***Pearson (r)*** | ***P-value*** | ***Inflammatory marker*** | ***Pearson (r)*** | ***P-value*** |
| CD68 | 0.81 | <0.0001 | CD68 | 0.61 | 0.03 |
| CD86 | 0.80 | <0.0001 | CD86 | 0.80 | 0.003 |
| TNF-α | 0.50 | 0.03 | TNF-α | 0.60 | 0.04 |
| CD11c | 0.97 | <0.0001 |  |  |  |
| CD163 | 0.73 | 0.0006 |  |  |  |
| TLR2 | 0.51 | 0.03 |  |  |  |
| MyD88 | 0.70 | 0.001 |  |  |  |
| CXCL-8/IL-8 | 0.90 | 0.0001 |  |  |  |
| **Non-diabetic Lean** | | | **Diabetic Lean** | | |
| ***Inflammatory marker*** | ***Pearson (r)*** | ***P-value*** | ***Inflammatory marker*** | ***Pearson (r)*** | ***P-value*** |
| None |  |  | None |  |  |

**Additional file 4. Within-group correlations of TLR8 expression with other inflammatory markers in the adipose tissue**
